# How Bacteria Cope with Oxidative Stress Induced by Cadmium: Volatile Communication Is Differentially Perceived among Strains

**DOI:** 10.3390/antiox13050565

**Published:** 2024-05-03

**Authors:** Paulo Cardoso, Ricardo Pinto, Tiago Lopes, Etelvina Figueira

**Affiliations:** 1 Department of Biology, University of Aveiro, 3810-193 Aveiro, Portugal; pjcardoso@ua.pt (P.C.); rl.pinto@ua.pt (R.P.); tslopes@ua.pt (T.L.); 2 CESAM—Centre for Environmental and Marine Studies, University of Aveiro, 3810-193 Aveiro, Portugal

**Keywords:** metals, volatile organic compounds, *Rhizobium*, biochemical parameters, bacteria–bacteria interactions

## Abstract

Soil is an environment with numerous niches, where bacteria are exposed to diverse conditions. Some bacteria are exposed earlier than others to pressure, and the emission of signals that other bacteria can receive and perceive may allow a better response to an eminent stimulus. To shed light on how bacteria trigger their response and adapt to changes in the environment, the intra- and interspecific influences of volatiles on bacterial strains growing under non-stressed and cadmium-stressed conditions were assessed. Each strain was exposed to its volatiles emitted by cells growing under different conditions to test whether the environment in which a cell grows influences neighboring cells. The five genera tested showed different responses, with *Rhizobium* displaying the greatest influence. In a second experiment, 13 strains from different genera were grown under control conditions but exposed to volatiles released by Cd-stressed *Rhizobium* cells to ascertain whether *Rhizobium*’s observed influence was strain-specific or broader. Our results showed that the volatiles emitted by some bacteria under stress are differentially perceived and translated into biochemical changes (growth, alteration of the antioxidant response, and oxidative damage) by other bacteria, which may increase the adaptability and resilience of bacterial communities to environmental changes, especially those with a prooxidant nature. Cadmium (Cd) contamination of soils constitutes a risk to the environment and human health. Here, we showed the effects of Cd exposure on bacteria and how volatile communication influences the biochemistry related to coping with oxidative stress. This knowledge can be important for remediation and risk assessment and highlights that new biological features, such as volatile communication, should be considered when studying and assessing the impact of contaminants on soil ecosystems.

## 1. Introduction

The environment is constantly influenced by natural and anthropogenic factors, causing changes in the conditions in which organisms live and impact communities [1,2]. Organisms such as bacteria may undergo transcriptional alterations that will have an impact on their metabolism and survival [3,4]. However, some bacteria within a community, known as persisters, can survive in unfavorable conditions by escaping the effects imposed by constraints without undergoing genetic change [5]. The persistence of bacteria is a current topic that has been extensively explored in the context of human health and is often associated with antibiotic resistance [5,6]. Both mechanisms imply changing the diversity of bacterial communities, replacing those that cannot survive these conditions with those that adapt or persist in impacted environments.

Agricultural systems are particularly affected by soil mobilization, monoculture farming, and excessive use of agrochemicals and fertilizers [7,8,9]. The application of fertilizers of chemical origin has allowed an increase in productivity and the supply of food for a growing population [10]; however, the levels of application sustaining high productivity have several negative impacts [11,12]. One of the side effects is the increased cadmium (Cd) concentration in phosphate fertilizers [13,14]. In soil, Cd concentration can vary between 0.07 and 1.1 mg Kg^−1^, but in agricultural soils can reach ≈100 mg kg^−1^ soil [15]. Numerous methodologies have been developed (physical, chemical, biological, and biotechnological) to mitigate biodiversity alterations and increase the resilience of bacterial communities to impacted environments, such as metal contamination; however, the effectiveness of these methods often falls short of expectations [16,17,18,19].

There are few sterile terrestrial ecosystems, as there are survivors who successfully adapt to extreme and changing conditions [20]. In this way, a deep knowledge of the strategies used by communities to adapt to new conditions could be the basis for adopting more efficient mitigation strategies. Due to the high heterogeneity and complexity of soils, microsites with different conditions are present, which results in organisms, especially small ones such as bacteria, in the same soil being differently exposed to a constraint [21]. This fact certainly affects bacterial survival and may contribute to explaining, beyond persisters, the fact that bacteria with different levels of tolerance are present in contaminated soil. However, this tolerance variability may be based on other assumptions that need to be unveiled.

Bacteria are capable of releasing a wide variety of volatile organic compounds (VOCs) [22] with potential roles in microbe–microbe interactions [23]. These molecules present low molecular weight (<300 Da) and high vapor pressure (0.01 kPa at 20 °C), allowing a rapid evaporation and diffusion to the environment [22]. Several bacterial VOCs have already been identified and have various capabilities ranging from antibacterial activity, and resistance against biotic and abiotic factors, to promoting plant growth [24,25,26]. Communication is important when microbes live freely in the environment and may contribute to establishing an equilibrium between microbes [27]. It is particularly important to elucidate the role of interspecific communication as a mechanism to increase the survival of bacteria challenged with stress. Knowledge of bacterial crosstalk can have implications for bacterial ecology [28]. Bacterial volatiles can thus emerge as a tool to influence the bacterial stress response, since changes in the transcriptome of other bacteria, namely, important rhizobacteria, can be induced by bacterial volatiles [29].

However, the quantitative and qualitative modulation of the bacterial volatile profile in adverse conditions remains largely unknown. Since VOCs are molecules that move in the air, could changes in their production represent messages perceived at a distance, a capacity common to bacteria? And would the message be perceived similarly by different members of a bacterial community?

To answer the first question (intraspecific influence of VOCs), five genera of bacteria were exposed to two different conditions (presence and absence of Cd) sharing the same atmosphere and growth in each condition compared to bacteria exposed to only one condition (no Cd or with Cd).

To answer the second question (interspecific influence of VOCs), the genus with the highest airborne influence was placed to grow in divided plates in the presence of Cd and the atmosphere shared with 13 bacterial genera growing without Cd. Growth and biochemical endpoints were determined for each genus and compared to the same genus without the volatile influence. The identification of the VOCs that may be inducing the responses was also carried out.

## 2. Materials and Methods

### 2.1. Bacterial Cultures

Thirteen strains of bacteria previously isolated from root nodules of different legume species [30] were used, including three strains of *Rhizobium* (Rz (KY491644), R1 (ON419188.1), R2 (MH236721.1)), *Paenibacillus* (B) (MH236742.1), *Pseudomonas* (P) (MH236649.1), *Flavobacterium* (F) (MH236743.1), *Erwinia* (E) (MH236654.1), *Herbaspirillum* (H) (MH236662.1), *Variovorax* (V) (MH236738.1), *Achromobacter* (Am) (MH236656.1), *Lysobacter* (L) (MH236701.1), *Acinetobacter* (A) (MH236719.1), and *Stenotrophomonas* (S) (MH236676.1). All bacteria were grown in yeast mannitol agar (YMA) and stored at 4 °C before the experiments.

### 2.2. Experimental Conditions

**Figure 1:**
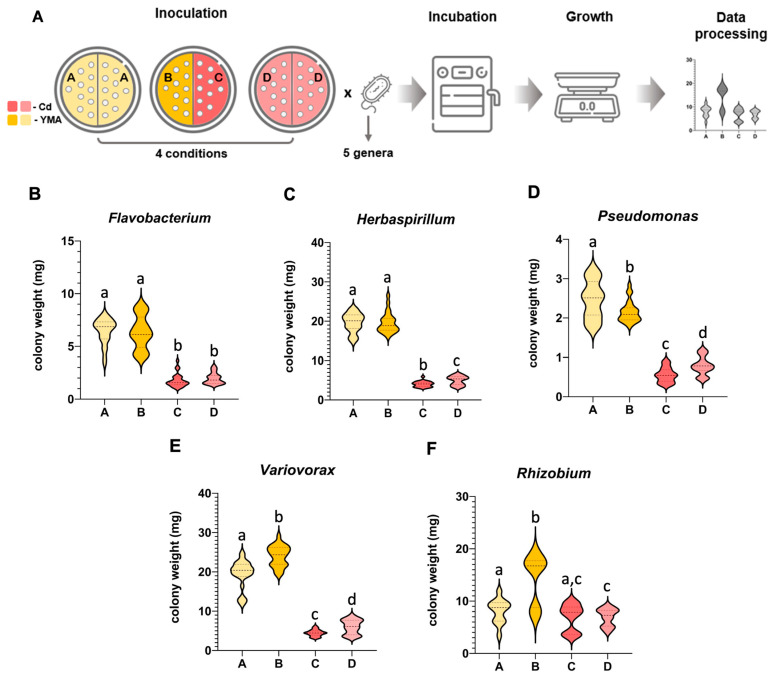
Intraspecific influence of bacterial volatiles upon colonies growing under different conditions: (**A**) Experimental design and procedure. A—cells grown in YMA and exposed to volatiles of cells also grown in YMA; B—cells grown in YMA and exposed to volatiles of cells grown in YMA containing Cd (100 μM); C—cells grown in YMA containing Cd and exposed to volatiles of cells grown in YMA; D—cells grown in YMA containing Cd and exposed to volatiles of cells grown in YMA containing Cd (100 μM). (**B**) Colony weight of the *Flavobacterium* strain. (**C**) Colony weight of the *Herbaspirillum* strain. (**D**) Colony weight of the *Pseudomonas* strain. (**E**) Colony weight of the *Variovorax* strain. (**F**) Colony weight of the *Rhizobium* strain. Violine plots were constructed with 24 replicates (3 to 5 replicates from 6 independent experiments). Different lowercase letters represent significant statistical differences (*p* < 0.05) among conditions for each strain.

Experiment 1: To evaluate the influence of volatiles released by each strain on the growth of cells of the same strain under two conditions (stressed and non-stressed cells), the first experimental design was set up (Figure 1A). Divided Petri plates with a central partition were used, allowing the exchange of volatiles from the two sides of the plate, as described by Farag and Kim [31,32]. Each side of the plate was inoculated with 10 colonies of a strain belonging to five different genera (*Flavobacterium* F, *Herbaspirillum* H, *Pseudomonas* P, *Variovorax* V, and *Rhizobium* Rz). Each strain was grown under four different conditions: A—grown in YMA and exposed to volatiles of cells also grown in YMA; B—grown in YMA and exposed to volatiles of cells grown in YMA containing Cd (100 μM); C—grown in YMA containing Cd (100 μM) and exposed to cells grown in YMA; D—grown in YMA containing Cd (100 μM) and exposed to cells grown in YMA containing Cd (100 μM). Six independent experiments, with three to five replicates (plates with 10 colonies on each side), were performed. The inoculated plates were sealed with two layers of parafilm and incubated for 3 days at 26 °C. After the incubation period, the bacterial colonies were collected in a 2 mL microtube and weighed to evaluate the influence of the volatiles on bacterial growth.

**Figure 2:**
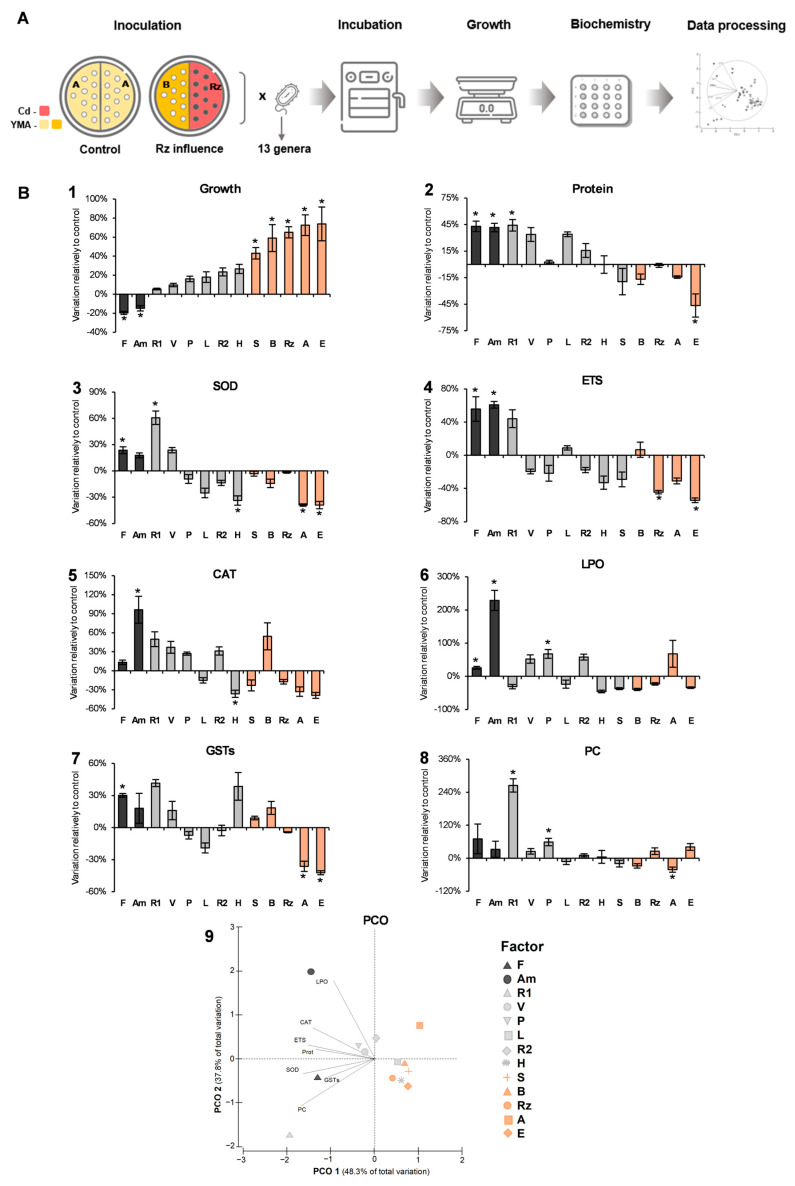
Influence of bacterial volatiles upon growth, oxidative damage, and antioxidant mechanisms under different conditions: (**A**) Experimental design and procedure. A—cells grown in YMA and exposed to volatiles of cells also grown in YMA; B—cells grown in YMA and exposed to volatiles of *Rhizobium* cells grown in YMA containing Cd (100 μM). (**B**) relative growth and biochemical parameters evaluated of strains exposed to volatiles released by E20-8 strain under cadmium stress (100 μM). 1—relative growth of 13 strains compared to control (no cadmium exposure); Biochemical parameters evaluated compared to control (no cadmium exposure): 2—Protein content, 3—Superoxide Dismutase (SOD) activity, 4—Electron Transport System (ETS) activity, 5—Catalase (CAT) activity, 6—Lipid Peroxidation (LPO), 7—Glutathione S-Transferase (GST) activity, 8—Protein Carbonylation (PC); 9—Principal Coordinate Ordination (PCO) of the biochemical determinants for each strain (Pearson correlation vectors were imposed: lipid peroxidation (LPO); Catalase activity (CAT); Electronic Transport System activity (ETS); Protein content (Prot); Superoxide Dismutase activity (SOD); Glutathione S-Transferase activity (GST); Protein Carbonylation (PC)). For growth, values are the means of 12 replicates (3 replicates from 4 independent experiments) + standard error. For biochemical analysis values are means of four replicates + standard error. Asterisks indicate significant differences (*p* < 0.05) compared to control.

Experiment 2: To assess the influence of volatile compounds released by *Rhizobium* Rz strain under Cd stress on other bacteria genera (*Rhizobium* (3 strains—Rz, R1, and R2), *Paenibacillus* (B), *Pseudomonas* (P), *Flavobacterium* (F), *Erwinia* (E), *Achromobacter* (Am), *Herbaspirillum* (H), *Variovarax* (V), *Lysobacter* (L), *Acinetobacter* (A), and *Stenotrophomonas* (S)), a second experimental design was set up (Figure 2A). One side of a divided plate containing YMA supplemented with 100 μM Cd was inoculated with *Rhizobium* Rz, and the other side containing only YMA medium was inoculated with one of the 13 strains. For the control condition, two sides of the plate contained YMA and were inoculated with one of the 13 strains. Four independent experiments were performed, with three replicates each. Plates were incubated, and growth was determined as described in Experiment 1. The bacterial colonies were stored at −80 °C for further analysis.

### 2.3. Biochemical Analysis

#### 2.3.1. Extraction

Samples were mixed with 1 mL of sodium phosphate buffer (50 mM sodium dihydrogen phosphate monohydrate, 50 mM disodium hydrogen phosphate dihydrate; 1 mM ethylenediaminetetraacetic acid disodium salt dihydrate (EDTA), 1% (*v*/*v*) Triton X-100; 1% (*v*/*v*) polyvinylpyrrolidone (PVP), 1 mM dithiothreitol (DTT), pH 7.0), and lysed using an ultrasound probe (Vibra Cell Ultrasonic Processor, Sonics, Newtown, USA ) for 60 s, applying a continuous cycle with 50% amplitude. Samples were centrifuged at 10,000× *g* for 10 min, and the supernatant was collected and immediately used or frozen at −80 °C for protein, protein carbonylation, LPO content, and SOD, CAT, and GST activity. For ETS, the procedure was similar, but samples were centrifuged at 3000× *g* for 3 min.

#### 2.3.2. Protein Content

Protein content was measured by the method described by Robinson [33]. In a 96-well plate, 50 µL of the supernatant was mixed with 250 µL of the biuret reaction and incubated in the dark for 10 min at room temperature. Absorbance was measured at 540 nm, and bovine serum (BSA) was used as standard (1–20 mg/mL). The results are expressed in milligrams per gram of fresh weight (mg BSA/g FW).

#### 2.3.3. Protein Carbonylation

The method described by Mesquita [34] was used in this study. In a 96-well plate, 125 µL of supernatant was mixed with 120 µL of 2,4-dinitrophenylhydrazine (DNPH) 10 mM and incubated for 10 min at room temperature. Then, 60 µL of sodium hydroxide (NaOH) 6 M was added and the mixture was incubated for more 10 min at room temperature. Absorbance was measured at 450 nm with 10 s of agitation before reading. The concentration was determined using the molar extinction coefficient of protein carbonyl-DNPH hydrazine (Ɛ = 22,308 M^−1^ cm^−1^), and the results were expressed in nanomoles per gram of fresh weight (nmol/g FW).

#### 2.3.4. Lipid Peroxidation

Cell membrane damage was estimated based on the lipid peroxidation level using the method described by Buege [35]. In a 2 mL microtube, 50 µL of supernatant was mixed with 200 µL of 0.5% thiobarbituric acid (TBA) and 150 µL trichloroacetic acid (TCA) and incubated for 25 min at 96 °C. The reaction was stopped by placing microtubes on ice. The cooled mixture was pipetted into a 96-well plate, and the absorbance was measured at 532 nm. The malondialdehyde (MDA) extinction coefficient (ε = 1.56 × 10^5^ M^−1^ cm^−1^) was used to calculate lipid peroxidation, and the results were expressed in nmoles of MDA equivalents per gram of fresh weight (nmol MDAeq/g FW).

#### 2.3.5. Superoxide Dismutase

Superoxide dismutase activity was evaluated using the methodology described by Beauchamp [36]. In a 96-well plate, 25 µL of supernatant was mixed with 25 µL of xanthine oxidase and 250 µL of reaction buffer containing nitroblue tetrazolium (NBT). The samples were incubated for 20 min at room temperature in an orbital shaker (150 rpm), and the absorbance was measured at 640 nm. Results were estimated in units of enzymatic activity (U), referring to a 50% reduction of NBT, and expressed in U per milligram of fresh weight (U/g FW).

#### 2.3.6. Catalase

The methodology described by Johansson [37] was used to measure catalase activity. In a 96-well plate, 25 µL of supernatant was mixed with 125 µL reaction buffer (potassium phosphate 50 mM, pH7.0, EDTA 1 mM Triton X-100 1%), 37.5 µL methanol, and 25 µL hydrogen peroxide (H_2_O_2_) 35.28 mM, and incubated for 20 min at room temperature in an orbital shaker (150 rpm). After, 37.5 µL of potassium hydroxide (KOH) 10 M and 37.5 µL purpald 34.2 mM were added and incubated for 10 min under the same conditions. Finally, 12.5 µL potassium periodate 65.2 mM was added and incubated for 10 min. The absorbance was measured at 540 nm and formaldehyde 4.24 mM was used as standard (2.5–20 µg/mL). Results are expressed in units of enzymatic activity (mU) per g of fresh weight (mU/g FW).

#### 2.3.7. Glutathione S-Transferases

The methodology proposed by Habig [38] was used to determine the glutathione S-transferase activity. In a 96-well plate, 100 µL of supernatant was mixed with 200 µL reaction solution (potassium phosphate buffer 0.1 M, pH 6.5, reduced glutathione (GSH) 10 mM, and 1-chloro-2,4-dinitrobenzene (CDNB)) 60 mM. Absorbance was measured at 340 nm for 5 min at intervals of 15 s with agitation for 6 s before reading the absorbance. The results are expressed in milliunits of enzymatic activity (mU) per gram of fresh weight (mU/g FW).

#### 2.3.8. Electron Transport System

The methodology proposed by De Coen [39] was used to assess the activity of the electron transport system. In a 96-well plate, 35.7 µL of supernatant was mixed with 107 µL Tris buffer (0.13 M Tris-HCl, 0.3% (*v*/*v*) Triton X-100, pH 8.5), 35.7 µL NADH 1.7 mM and 35.7 µL NADPH 250 µM, and 71.4 µL p-iodonitrotetrazolium (INT) 8 mM. The reaction was initiated by the addition of INT, and the absorbance was measured at 490 nm for 10 min at intervals of 25 s. Results were calculated using the formazan extinction coefficient (Ɛ = 15,900 M^−1^ cm^−1^) and expressed in nanomoles per minute per gram of fresh weight (nmol/min/g FW).

### 2.4. Volatiles Released by Rhizobium sp. E20-8

Headspace-solid phase microextraction (HS-SPME) was used for the extraction and gas chromatography coupled with mass spectrometry (GC-MS) for the detection and identification of volatile compounds released by *Rhizobium* sp. E20-8. The methodology proposed by Farag [40] was adopted with some modifications. The strain was grown for three days at 26 °C in plates containing YMA medium or YMA supplemented with 1 mM Cd. After incubation, the bacterial content was collected using a stainless-steel spatula into 12 × 32 mm, 2 mL screw vials for chromatography with screw caps, and polytetrafluoroethylene (PTFE)/silicon septa. Each vial contained 300 mg of bacterial colonies. Care was taken to avoid the collection of culture medium. Then, 10 μL of an aqueous solution containing 1 μg of (Z)-3-Hexenyl acetate (Sigma-Aldrich, St. Louis, USA, CAS 3681-71-8) was spiked into each vial before extraction to be used as internal standard, using a Hamilton syringe. The internal standard was freshly prepared by diluting the compound in Milli-Q water. A stable flex divinylbenzene/carboxen/PDMS 50/30 μM 1 cm fiber (Supelco, St. Louis, USA) was inserted into the vial. Extraction was performed by placing each vial in a water bath (50 °C) for 30 min. For each condition (YMA and YMA supplemented with Cd), four vials were prepared and analyzed using GC-MS. The GC-MS system was composed of an Agilent Technologies 7890B GC System coupled to an Agilent Technologies 5977A MS. Tekno TRB-5MS (Teknokroma, Barcelona, Spain) was used in this experiment. The GC-MS method was based on Farag [40] The main difference was the MS source temperature, which, in our case, was 230 °C instead of 200 °C. The ratios of peak height and the height of the internal standard were calculated, and identification of the compounds was performed by comparison of spectra with spectra from the NIST14 database and comparing the Kovats retention index with values from the literature, which were calculated by running a series of C8-C20 n-alkanes under the same conditions as the samples).

### 2.5. Data Analysis

Data from bacterial growth and biochemical parameters were subjected to hypothesis testing by Permutational Multivariate Analysis of Variance (PERMANOVA) using Primer 6 & PERMANOVA+. Monte Carlo permutations (9999) were used to run the PERMANOVA tests. The pseudo-F values in the main tests were evaluated for significance, and when *p* < 0.05, pairwise comparisons were performed. The null hypothesis tested was that there were no significant differences between the control and tested conditions. Principal component analysis of GC-MS data was performed using Metaboanalyst 5.0 [41]. The ratio of peak height/peak height of the internal standard was log-transformed (base 10) and auto-scaled (mean-centered and divided by the standard deviation of each metabolite).

## 3. Results

### 3.1. Mutual Airborne Influence of a Bacterial Strain Exposed to Different Conditions Varied among Bacterial Genera

The airborne influence that a strain may impose on itself when experiencing different conditions was evaluated by growing the same bacterial strain in divided Petri plates (shared atmosphere) with conditions differing on each side of the plate (without Cd, condition B and with Cd, condition C) and the growth compared to the same bacterial strain growing under the same conditions on both sides of the plate (without Cd, condition A and with Cd condition D). The experiment included five bacterial genera to ascertain whether the influence was a common feature among bacteria or if it was species-dependent (Figure 1A).

The *Flavobacterium* strain was poorly influenced by the volatiles produced by cells growing under different conditions (Figure 1B). In the absence of Cd, the growth of bacteria in condition B was not significantly different from condition A, although a higher heterogeneity among colony size was noted due to a higher proportion of smaller colonies, reducing the average size compared to condition A. In the presence of Cd, the growth between C and D conditions was also not significant, but a higher number of smaller colonies and a lower number of larger colonies led to a slightly smaller average colony size in C compared to D.

*Herbaspirillum* strains grown in the absence of Cd but influenced by the atmosphere of Cd-exposed bacteria (condition B) were not significantly affected by the volatiles released by Cd-stressed cells, but the reverse was not true, with cells growing in the presence of Cd (C) being negatively affected by the volatiles produced by cells from condition B (Figure 1C).

Volatiles from *Pseudomonas* (Figure 1D) and *Variovorax* (Figure 1E) induced significant differences in the growth of colonies in both plate compartments. In condition C, the effect was negative for both genera, with colonies being smaller in C than in D. *Pseudomonas* colonies also grew less in B than in condition A, but in *Variovorax*, colony size was larger in B than in A.

The most positive airborne influence was noticed in the *Rhizobium* strain (Figure 1F), with colonies in B being significantly larger (75%) than in A, and with some colonies in condition C showing higher growth than in condition D, and that a high heterogeneity of sizes (larger and also smaller colonies) did not show a significant difference from D, but that was also not significantly different from A.

### 3.2. The Volatiles Produced by Rhizobium Grown in Cd Influenced Distinctly the Growth of 13 Bacterial Strains

The universality among bacteria of the positive influence of the volatiles produced by *Rhizobium* E20-8 (Rz in experiment 1) under Cd stress was investigated by growing in the absence of stress 13 bacterial strains belonging to 11 genera, but receiving the airborne influence of the volatiles released by Rz exposed to Cd by a shared atmosphere in divided plates (Figure 2A). The volatile influence was evaluated through growth and biochemical endpoints relatively to control (Figure 2B).

Two strains exposed to *Rhizobium* volatiles (*Flavobacterium* and *Achromobacter*) grew significantly less (black bars), seven strains were not significantly influenced (grey bars), and five strains showed significantly higher growth (orange bars) compared to control. Of the three strains belonging to the genus *Rhizobium* (one was Rz), two were positively and significantly influenced (Rz and R2), but R1 growth was not influenced.

### 3.3. The Antioxidant and Biotransformation Mechanisms Seem to Be Induced in Strains That Grew Less and to Be Reduced in Those That Grew More in the Presence of Rhizobium Volatiles (Rz VOCs)

The three enzymes assayed, superoxide dismutase (SOD), catalase (CAT), and glutathione-S-transferases (GSTs) showed a trend to decrease activity with the increase in strains growth caused by the Rz VOCs (Figure 2B). In GSTs, increases higher than 30% were noticed in three strains (*Flavobacterium*, *Rhizobium* R1, and *Herbaspirillum*), but only *Flavobacterium* showed a significant increase, and in the two strains with higher growth (*Acinetobacter* and *Erwinia*), a significant decrease was observed. A 100% increase in CAT activity was observed in the *Achromobacter* strain exposed to Rz VOCs. Most of the strains with growth not significantly influenced by Rz VOCs also increased CAT activity between 20 and 50%, although not significantly, but in the *Herbaspirillum* strain CAT activity decreased significantly. Most strains positively influenced by Rz VOCs showed a non-significant decrease in activity (12% to 32%). SOD activity increased in the strains with lower growth (*Flavobacterium*, *Achromobacter*, *Rhizobium* R1, and *Variovarax*) and did not vary or decreased in others, significantly in *Herbaspirillum*, *Acinetobacter*, and *Erwinia*.

### 3.4. Changes in Cell Metabolism Seem to Be Inversely Related to the Influence of Rz VOCs on Growth

Protein content increased significantly in the three strains with lower growth (*Flavobacterium*, *Achromobacter*, and *Rhizobium* R1) and decreased significantly in the strain most positively influenced by Rz VOCs (Figure 2B). The same trend was perceived in the electron transport chain (ETS) activity, with the *Flavobacterium* and *Achromobacter* strains increasing significantly and *Rhizobium* Rz and *Erwinia* significantly decreasing ETS activity.

### 3.5. Strains with Lower Growth Evidenced Higher Membrane and Protein Damage

Lipid peroxidation (LPO) significantly increased in the two strains that grew less in the presence of Rz VOCs and in one strain (*Pseudomonas*) whose growth was not influenced. Most strains with growth increased by Rz VOCs decreased LPO levels, although not significantly (Figure 2B). Protein carbonylation (PC) followed a similar trend, with two strains whose growth was less affected by Rz VOCs (*Rhizobium* R1 and *Pseudomonas*) showing higher increases in PC, and most positively influenced strains evidencing small variations in PC levels, except strain *Acinetobacter*, which presented a significant decrease.

### 3.6. Most Biochemical Changes Were Highly Correlated with Strains That Grew Less under the Influence of Rz VOCs

Principal coordinates analysis (PCO) shows that the abscissa axis (PCO1) is responsible for 48.3% variation, separating strains positively from those negatively influenced by Rz VOCs, based on their different biochemical responses (Figure 2B). The ordinate axis (PCO2) is responsible for 37.8% variation, separating strains by the biochemical mechanisms activated. Biochemical parameters were mostly correlated with strains that grew less by the influence of Rz VOCs, but different strategies were noticed. *Achromobacter* strain induced CAT activity, but membranes were still vulnerable to VOCs, with LPO being highly correlated with this strain. GSTs and SOD activities were highly correlated with *Rhizobium* R1 and *Flavobacterium* strains and so was protein carbonylation.

### 3.7. Exposure to Cd Alters Rhizobium Volatilome

**Figure 3:**
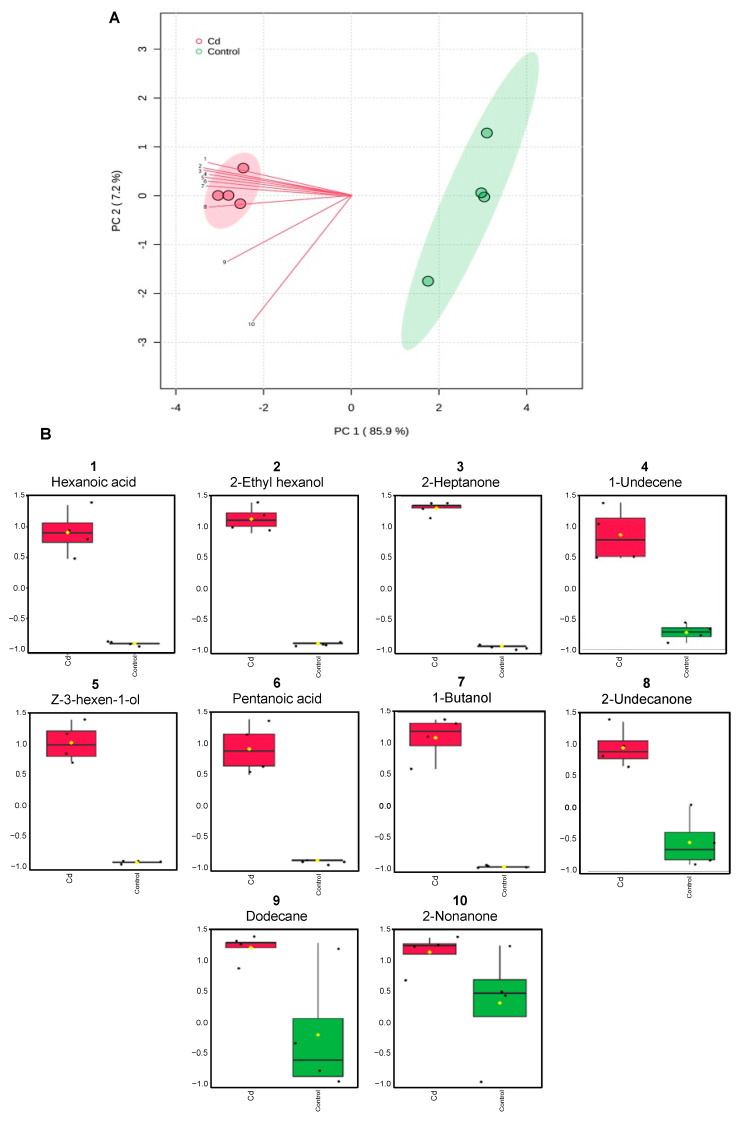
Volatiles released by *Rhizobium* sp. E20-8 under control and Cd-exposed conditions: (**A**) Principal Component Analysis (PCA). (**B**) Boxplots of the detected compounds. Peak intensity of compounds from 4 replicates (vails containing bacterial cells grown under control or Cd-exposed conditions) was log-transformed.

HS-SPME and GC-MS of the volatiles released by *Rhizobium* Rz grown in the control and in the presence of Cd allowed the identification of 10 volatile compounds. The exposure to Cd led to changes in the volatilome of the bacterial strain, as evidenced in Figure 3A. Samples of control and Cd-exposure were separated by component 1 of the principal component analysis, which explained 85.9% of the variation. For most compounds, emissions increased in cells exposed to Cd (Figure 3B).

## 4. Discussion

Volatiles are involved in the interactions between bacteria and other organisms (including other bacteria) [42]. Tyc [43] reported that the growth of bacterial strains was either stimulated, inhibited, or unaltered when exposed to volatiles emitted by bacteria of different species. *Bacillus subtilis* and *Escherichia coli* were capable of inhibiting competitor biofilms [44]. *Burkholderia ambifaria* produced volatiles with high bioactivity independently of the environment where they were produced (clinical environment, rhizosphere, or plant root) [45], showing that the origin of the bacterial isolates is likely not a crucial factor behind interactions and producing a general effect. The authors in [46] also observed a general trend of bacteria influencing their peers, with 40% of cocultures containing at least one strain facilitating the other’s growth. On the contrary, mechanisms such as necrosignaling, which was detected in both Gram-negative and Gram-positive bacteria, influenced swarming resistance to antibiotics and appeared to be species-specific [47].

The aforementioned studies address the influence between bacteria of different species, yet the interactions that may occur between the same bacteria under different conditions, which often occur in soil microheterogeneity, are virtually unknown. This was one of the impetuses for this study. To test whether changes that occur in a bacterial cell can influence other cells of the same bacterial strain growing under different conditions (non-stressed and Cd-stressed), whether this influence can be transient, and whether it is a universal feature. Our results confirmed that stress conditioned the cells directly exposed to Cd, but also influenced nearby bacteria of the same strain not exposed to Cd. It was shown that this influence can be volatile, since the shared atmosphere of divided plates with different Cd conditions at each side induced differences in growth. This volatile influence is not a common characteristic among bacteria, since from the five bacterial strains tested, positive, negative, and no influence on non-stressed cells were observed. Among the strains tested, *Rhizobium* E20-8 was the strain whose volatiles released by Cd-stressed cells had the most positive effect on non-Cd-stressed cells. The ability of different strains belonging to 11 bacterial genera to perceive the volatiles released by Cd-stressed *Rhizobium* E20-8 showed distinct responses. Some strains benefited from E20-8 volatiles (higher growth or lower oxidative stress), while others were impaired or not influenced. These results are indicative of the heterogeneity of the effects of volatiles, which depend on the receptor bacteria, and are also evidence of the bacterial volatiles importance as a mechanism of interaction between bacteria, intra and interspecifically. Several bacterial volatiles have been linked to the stimulation of bacterial stress resistance, namely, 2,3-butanedione, glyoxylic acid, hydrogen sulfide, nitric oxide, ammonia, indole, trimethylamine, 1-methylthio-3-pentone, and 2-amino-acetophenone [48].

Our study provided additional compounds that may be inducers of stress resistance or stress biomarkers, which were released by *Rhizobium* E20-8 cells facing Cd stress and absent in control conditions. Indeed, the analysis of the *Rhizobium* E20-8 volatilome revealed volatiles, such as 1-butanol, 2-nonanone, and 1-undecene, which may be linked to the effects of the E20-8 strain on itself and other bacterial genera (Figure 3).

1-Undecene was reported to be responsible for the in vitro inhibition of *Legionella pneumophila* growth by *Pseudomonas fluorescens* [49]. *Bacillus subtilis* and *Escherichia coli* inhibited competitor biofilms by releasing 1-butanol and 2-nonanone [44]. In our study, the shift of *Rhizobium* E20-8 volatilome induced by Cd evidenced that the same compounds (1-butanol, 2-nonanone, and 1-undecene) may also be used to signal Cd stress and evidence the activity of the same compounds towards different stresses. Due to their ability to work as inhibitors of competitive biofilms, we can hypothesize that these compounds may be released by *Rhizobium* E20-8 as a mechanism of competition under stressful conditions. However, this mechanism proved to be effective against some strains but not against others. Moreover, our study showed that in the presence of the volatiles emitted by *Rhizobium* E20-8, effects could be observed not only for cells of the same strain but also across strains and genera, with changes in growth, antioxidant enzyme activity, metabolism, and damage. These compounds were involved in oxidative stress response, and the blend of volatiles produced by *Rhizobium* E20-8 influenced the oxidative status of each strain differently. Antioxidant and biotransformation mechanisms were induced in strains negatively affected by volatilome changes and decreased in the positively influenced strains. Responses at the subcellular level are important since mitigation of reactive oxygen species is crucial for bacterial survival [50]. Previous research has shown that growth, antibiotic production, and gene expression of the bacterium *Pseudomonas fluorescens* Pf0-1 were influenced by the volatiles released by four phylogenetically different strains (*Collimonas pratensis*, *Serratia plymuthica*, *Paenibacillus* sp., and *Pedobacter* sp.) and that the bacterial volatiles induced chemotactic motility and oxidative stress responses and growth of *P*. *fluorescens* was stimulated [51]. The generation of reactive oxygen species is involved in lethal attacks between interspecies competition, both in contact-dependent and contact-independent interactions [52]. Indeed, the strains evidencing higher oxidative effects generated by E20-8 volatiles grew less, evidencing higher susceptibility to this type of interaction. The released volatiles produced as a result of stress also impacted the metabolism of positively influenced receptor cells and may be sensed as warning signals, as suggested by previous results [53], inducing changes and triggering in advance the response of non-stressed cells to eminent stress.

The present study shed light on the effects that volatiles may have on the biochemical and metabolic status of different bacterial cells. By studying the interspecific interactions between strains of different genera, and reporting the effects not only on antioxidant machinery but also on metabolism and damage, our study contributes to advance knowledge on bacterial interactions, since most bacteria–bacteria studies are performed using two microbial cultures usually belonging to different species [54] and are often limited to changes in growth. These results also point out that volatile communication is interpreted differently among members of a community, contributing to changes in its diversity and abundance. Communication in microbial communities is important to facilitate survival and evidence is accumulating on the ability of bacterial cells to sense and respond to volatiles, with influence (stimulation or inhibition) in antibiotic resistance, biofilm formation, and virulence [42]. Volatile emissions by microbes have been reported to increase, in the soil, when there is a lower diversity of microbes [55], suggesting that the volatile communication between soil microorganisms might be a mechanism on which communities rely for equilibrium. Our results bring evidence that in a microbial community, the response of an organism to a change (e.g., metal stress) not only corresponds to the direct effect of the stressor on the organism but is also influenced by other organisms, helping to understand why responses in the laboratory are often not replicable in the environment. These findings are fundamental to a better understanding of bacterial ecology and how they respond to stress conditions as a community, from agricultural to clinical standpoints. However, questions remain on how bacterial VOCs affect bacterial growth and biochemistry. Would exposure to different stresses induce the same or different influences? Exposing bacteria to individual VOCs could clarify the specific effects of each compound. Metabolomics and transcriptomics approaches could bring information to assess how the same blend of VOCs has different effects in different bacteria.

## 5. Conclusions

The present study shows that some strains (here, denominated as “inducer” bacteria) have a higher ability, through volatiles, to influence the response of others. These bacteria may play an important ecological role by influencing the growth and metabolism (particularly the antioxidant response) of neighboring bacteria, helping define the response of a microbial community facing stresses, especially those changing cell redox status, and expectably making it more resilient to new conditions.

The identification of strains (“inducer” bacteria) and the volatiles they produce can be used as a biotechnological approach for contaminated sites, increasing the resilience of the existing bacterial community to contamination and restoring the activities of native soil microflora such as nutrient cycling, nitrogen fixation, improvement of soil structure and degradation, and the detoxification of pollutants in the soil. This way, the recovery of contaminated sites can be boosted, rendering remediation strategies more efficient. The negative influence that “inducer” bacteria and their volatiles have on some bacteria also demonstrates the potential to be used in a clinical context, by sensitizing pathogenic bacteria and making them more vulnerable to antibiotics, which can be important in the current context of increasing resistance to antibiotics.

## Data Availability

The data presented in this study are available on request from the corresponding author.
